# Improving the Quality and Standardization of Operative Notes in a Tertiary Regional ENT Department: A Closed-Loop Audit

**DOI:** 10.7759/cureus.63398

**Published:** 2024-06-28

**Authors:** Mustafa Qasem, Omar Qasem, Nikoleta Skalidi

**Affiliations:** 1 Otolaryngology - Head and Neck Surgery, Liverpool University Hospitals NHS Foundation Trust, Liverpool, GBR; 2 Otolaryngology - Head and Neck Surgery, University of Jordan Hospital, Amman, JOR

**Keywords:** audit, surgery, quality improvement, ent, operative note

## Abstract

Introduction: Comprehensive and accurate documentation is paramount in ensuring patient safety, and continuity of care, and casts the foundation for auditing practice and creating research. Unfortunately, a lack of documentation has been reported globally. This study aims to challenge current practices and ensure high-quality documentation.

Methodology: The study appraised 100 operation notes completed within a tertiary, regional ENT department against the published guidance. Following an inquiry into the findings, the operative note proformas were modified in alignment with the Royal College of Surgeons (RCS) published standards for the quality of operative notes. A further 100 operation notes were audited. Rates of compliance before and post implementing the intervention were compared. Non-parametric data were analyzed using Fischer’s exact test, with *P *< 0.05 considered to be statistically significant.

Results: Upon auditing of operative note completeness against the set criteria, the use of a comprehensive operative note proforma significantly refined the quality of documentation and adherence to the published standards (*P *< 0.00001).

Conclusions: This study displayed the lack of adherence to the RCS standards about the quality of operative notes within our center. The adoption of a modified proforma which incorporates the 18 criteria defined by the RCS resulted in improved conformance to published standards and a higher quality of documentation. This study corroborates the RCS framework as an effective tool in recognizing deficient practices and areas of improvement. Through compliance with published standards, a higher quality of documentation is attained, contributing to patient safety, clear continued communication, and support of clinical governance.

## Introduction

The provision of high-quality healthcare relies heavily on the accurate and prompt recording of clinical interactions [1]. Medical records act as the channel for communication within a healthcare institution, allowing for uninterrupted and continued care [2]. Moreover, clinical documentation serves as a precious resource for purposes of audit and research, contributing to the foundation of clinical governance [3].

Operative notes represent a comprehensive detail of surgical procedures, displaying key information that is requisite for the immediate postoperative care requirements of patients and long-term follow-up care [4]. Deficient operative notes can result in errors during postoperative care, endanger patient health, and prolong hospital admissions [5]. These avoidable issues highlight the value of proper documentation practices. An operative note is also regarded as a vital record of significant medicolegal relevance. Comprehensive and upkept notes constitute evidence that may be paramount in resolving legal disputes [6]. Categorically, accurate and comprehensive operative notes contribute to an elevated standard of patient care, which remains the fundamental aim of healthcare institutions [7].

The Royal College of Surgeons (RCS) guidelines, defined in *Good Surgical Practice* 2014 - domain 1.3 are revered as the standard for the quality of operative notes [8]. The guidelines outline 18 essential criteria for operative documentation, emphasizing accuracy and clarity. The guidance is applicable across various surgical disciplines, and adherence to the defined criteria denotes a commitment to advancing the quality of operative documentation. However, despite the published standards, operative recordkeeping has been reported globally to be lacking in completeness and precision [2,9-11]. This study aims to assess the adherence within our department to said guidelines and discern areas for improvement. We reflect upon our experience and propose recommendations for elevating documentation practice.

The lack of adherence to the published standards could be due to the lack of a proper proforma or checklist. Without a standardized template for operative notes, surgeons might overlook certain details required by the guidelines. Our study aims to investigate this theory by examining whether the use of a standardized proforma improves the adherence of notes to the RCS guidelines.

What is already known on this topic

The RCS has established guidance on the quality, comprehensiveness, and clarity of operative notes. However, poor documentation practices have been repeatedly demonstrated across various specialties.

What this study adds

A reflection upon the experience of a tertiary ENT department in auditing documentation practice against the RCS guidelines and the impact of adopting a digital proforma on the quality of operative notes.

How this study might affect research, practice, or policy

This audit underlines the importance of comprehensive operative note documentation and the merit of adopting the RCS Good Surgical Practice standards as a framework to appraise and improve current practice.

## Materials and methods

Study design and intervention

Ethical approval was acquired from the Clinical Audit Management team of Liverpool University Foundation Trust. The audit was registered under Project No. 12263, which is the ethical approval number. From April to May 2023, 100 consecutive operative notes on adult patients undergoing Otolaryngology operations were identified through ATMIS software (DXC Technology, Tysons, VA), the interface used by the trust to manage data relating to operations held. Analysis of operative notes was performed against the published standards, and a meeting was held within our department to gain insight into factors limiting the rate of adherence and to propose strategies for improvement. A modified operative note proforma was designed and circulated within the trust that incorporated the 18 criteria set out by the RCS guidance, imploring keyword prompts and Boolean yes/no options (Appendix). A further audit cycle was conducted during March 2024, through which an additional 100 consecutive operative records were reviewed and analyzed.

Data extraction

A structured framework was drafted to extract data apropos of the RCS guidelines for operative note documentation. Data describing the following 18 domains were assessed: (1) date and time; (2) elective/emergency; (3) surgeon and assistant; (4) anesthetist; (5) operative procedure; (6) incision design; (7) operative diagnosis; (8) operative findings; (9) problems/complications; (10) extra procedures; (11) details of tissue removed, added, or altered; (12) prostheses or implants added; (13) closure technique; (14) estimated blood loss; (15) antibiotic prophylaxis; (16) venous thromboembolism (VTE) prophylaxis; (17) postoperative instructions; and (18) signature. Each category within the sample retrieved was itemized as “Y” (1) or “N” (0). Data extraction was conducted by a single author (QM).

Outcomes

The principal outcome of this study was to assess the extent of compliance with established standards of operative note recordkeeping. The RCS Good Surgical Practice guidelines represented ideal practice. The secondary outcome was to compare the quality of documentation practice depending on the setting of the procedure, whether the operation was performed as an emergency or as part of an elective list.

Statistical analysis

Rates of compliance with the RCS standards both before and after implementation of the modified proforma were compared. Nonparametric data were analyzed using Fischer’s exact test, with *P *< 0.05 considered to be of statistical significance.

## Results

Engagement

Although the inertia to change in current documentation practice was expected to limit the effectiveness of our intervention, findings suggest a high uptake of the modified proforma; 99/100 operative notes were completed via the revised template. 

Compliance with RCS guidelines

Results of auditing operative notes both before and after modification of the operative note proforma are outlined in Table 1. Overall compliance with the RCS gold standards exhibited pronounced correction from 58.1% to 99.6% upon effecting the adapted proforma (*P *< 0.00001). Significant improvements in comprehensiveness and precision were displayed across the following categories: operative setting (emergency vs. elective), anesthetist involved in theatre, incision utilized, operative diagnosis, problems and complications encountered, additional procedures performed, details of tissue manipulated, prostheses and implants used, closure technique, estimated blood loss, antibiotic prophylaxis, and VTE prophylaxis (*P *< 0.05). Higher rates of adherence were also noted in the following two domains: name of surgeon and operative findings, albeit not amounting to statistical significance. No reduction in rates of compliance was observed across any category following the application of the revised proforma.

**Table 1:** Percentage of operative notes containing RCS criteria. ^*^P < 0.05. RCS, Royal College of Surgeons; VTE, venous thromboembolism

Category	Pre-intervention (%)	Modified proforma (%)		*P*-value
Date and time	100	100	1.0000	
Elective/emergency	16	99*	<0.0001	
Name of surgeon	96	100	0.1212	
Name of anesthetist	84	94*	0.0400	
Operative procedure	100	100	1.0000	
Incision	60	100*	<0.0001	
Operative diagnosis	76	99*	<0.0001	
Operative findings	98	100	0.4975	
Problems/complications	13	100*	<0.0001	
Extra procedures and reasoning	40	100*	<0.0001	
Details of tissue manipulated	87	100*	0.0002	
Prostheses/implants	15	100*	<0.0001	
Closure technique	60	100*	<0.0001	
Estimated blood loss	0	100*	<0.0001	
Antibiotic prophylaxis	0	100*	<0.0001	
VTE prophylaxis	0	100*	<0.0001	
Post operative instructions	98	100	0.4975	
Signature	100	100	1	

Secondary outcome

Data comparing the rates of compliance according to the context of operative note documentation (emergency vs. elective) are outlined in Table 2. The setting of documentation did not appear to significantly impact the extensiveness of recordkeeping, neither before nor after the establishment of the revised proforma (*P *= 0.887).

**Table 2:** Rates of compliance with RCS guidelines according to the setting of operation. RCS, Royal College of Surgeons

	Pre-intervention		Modified proforma	
	Elective	Emergency	Elective	Emergency
Number of operations	84	16	88	12
Overall compliance (%)	59.1	55.6	99.7	98.2

## Discussion

This study describes a significant improvement in the precision and comprehensiveness of operative notes upon adopting a proforma inspired by the RCS standards for Good Surgical Practice. Before this audit, our department used 12 procedure-specific proformas and one generic operative note for documentation of surgical proceedings within theaters. The first auditing cycle revealed notable shortcomings in the ability of current practice to comply with published standards. None of the operative notes retrieved contained all 18 criteria advised by the RCS, and no record of estimated blood loss, antibiotic prophylaxis, or VTE prophylaxis was included in any instance. An overall rate of compliance to established standards of 58.1% urged a considerable requisite for improvement.

Upon presenting the initial findings at the monthly audit meeting held by the Otolaryngology department within our trust, several constructive recommendations were proposed. The limiting factors hindering compliance were argued to be the absence of an aide memoire that would facilitate the inclusion of all required parameters, as well as a lack of formal education of the registrars and junior doctors on the essential content of an operative note [12,13]. The RCS guidelines are generic and are meant to apply to all surgical specialties. Certain criteria may have often been overlooked as they were felt to only marginally apply to the otolaryngology operations frequently held at our department. A significant proportion of the operations performed were pan-endoscopies that do not utilize an incision, and thus do not require closure. Implants were only used in very few operations, ordinarily limited to middle ear surgeries which comprise a fraction of the procedures held at our regional Head and Neck Center. Documentation concerning antibiotic and VTE prophylaxis is invariably included in the sign-in checklist before the start of every operation, which might elucidate their omission from the operative note records. It was also considered that documenting the absence of complications may not have been routine practice, and mention of perioperative complications was only made in cases where an unexpected incident arose.

The meeting was used as a venue to circulate the RCS guidelines amongst surgeons of all seniority levels (consultant, registrar, and senior house officer), and the proformas were redesigned to account for deficiencies in memory aids. Despite the cross-specialty design of the RCS standards, it was agreed that all 18 criteria would be included in the modified proformas, to ensure the highest quality of operative documentation. A running, biannual audit cycle was agreed upon to reinforce improvements in compliance. This is meant to accommodate for the rotary nature of the training programs, and the frequent changeover of junior-level surgeons working within the department.

The results of our study align with published literature [14-17]. The introduction of a revised proforma that encompasses the parameters directed by the RCS Good Surgical Practice introduces significant refinement in both the accuracy and completeness of operative notes. A tangible advantage was seen in the use of procedure-defined proformas as opposed to the generic template (*P *< 0.05). The 13 online documents were modified to include the 18 recommended criteria and additional two procedure-specific proformas were designed to aid in the authoring of surgical notes for commonly performed operations within our department: tracheostomy and tonsillectomy. Nonetheless, irrespective of whether a procedure-specific proforma or a generic template is used, an outlined online form allows for documentation that is comprehensive, legible, contemporaneous, and safe. Education on current best practices, and the value of maintaining high standards of record-keeping, complements adherence to established guidelines and allows for more dependable service provision [18].

Although not part of the primary aims of this study, the findings appear to further corroborate the RCS Good Surgical Practice as an effective tool in auditing an institution’s performance in operative note documentation against best practice. Results identified areas of deficient practice and accentuated areas where intervention is needed to augment documentation quality.

Limitations

This study was limited in its external validity. Albeit in a regional, tertiary center, the context of this project remained within a single department. The depth of detail described in the Materials and Methods section aims to increase the generalizability of the findings, allowing reproducibility in other specialty departments and healthcare settings.

## Conclusions

This audit underlines the importance of comprehensive operative note documentation and the merit of adopting the RCS Good Surgical Practice standards as a framework to appraise and improve current practice. The findings provide valuable insight into factors limiting adherence to documentation standards and present effective recommendations to address them. The study demonstrates significant adherence upon adoption of the modified proformas across all essential parameters and emphasizes the value of a higher quality of documentation towards clear continued communication, patient safety, protection against medical litigation, and support of clinical governance.

## Figures and Tables

**Figure 1:**
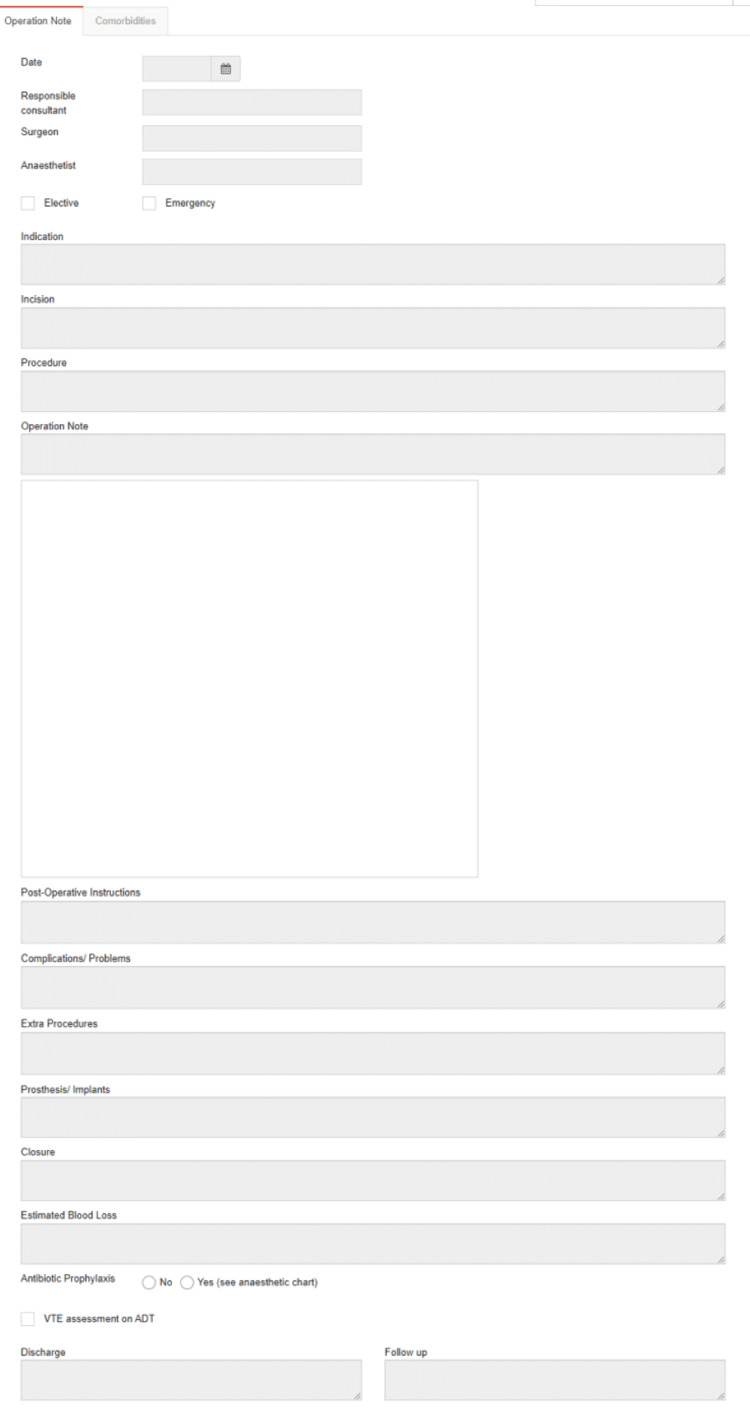
The generic modified operative note proforma containing the 18 criteria set out by the RCS. RCS, Royal College of Surgeons

## Appendices

Appendix
